# Acid-Sensing Ion Channels as Potential Therapeutic Targets in Neurodegeneration and Neuroinflammation

**DOI:** 10.1155/2017/3728096

**Published:** 2017-09-19

**Authors:** Audrey Ortega-Ramírez, Rosario Vega, Enrique Soto

**Affiliations:** Instituto de Fisiología, Benemérita Universidad Autónoma de Puebla, 14 sur 6301, CU, San Manuel, 72570 Puebla, PUE, Mexico

## Abstract

Acid-sensing ion channels (ASICs) are a family of proton-sensing channels that are voltage insensitive, cation selective (mostly permeable to Na^+^), and nonspecifically blocked by amiloride. Derived from 5 genes (*ACCN1–5*), 7 subunits have been identified, 1a, 1b, 2a, 2b, 3, 4, and 5, that are widely expressed in the peripheral and central nervous system as well as other tissues. Over the years, different studies have shown that activation of these channels is linked to various physiological and pathological processes, such as memory, learning, fear, anxiety, ischemia, and multiple sclerosis to name a few, so their potential as therapeutic targets is increasing. This review focuses on recent advances that have helped us to better understand the role played by ASICs in different pathologies related to neurodegenerative diseases, inflammatory processes, and pain.

## 1. Introduction

Extracellular acidification occurs in pathological situations, such as inflammation and brain ischemia, as well as under normal physiological conditions, such as neuronal activity and synaptic transmission. However, pH oscillations are closely regulated and confined to microdomains. Increased metabolism of carbohydrates produces a pH drop through products, such as lactic acid and CO_2_, activating HCO_3_^−^ and Na^+^/H^+^ exchangers that contribute to the regulation of pH [1].

The pH fluctuations affect many cellular processes, including enzymatic activity, membrane receptors, ion channel flow, and membrane transporters [2, 3]. Because pH is a strictly regulated variable in multicellular organisms, localized pH changes may constitute significant signals of cellular processes that occur in a cell or a group of cells. Extracellular pH changes that occur in microdomains are sensed through acid-sensing ion channels (ASICs), which are membrane channels that are specifically activated by protons (H^+^) and produce a large, inward, mostly Na^+^ current.

For accurate proton-mediated signaling, tight extracellular pH regulation is essential; failure of the pH buffering systems may lead to noisy activation of the system and no signal at all [4]. Recent works have shown that protons are mediators and ASICs are receptors in some synapses in the central nervous system (CNS) [5, 6]. The proton-mediated signaling mechanism was first demonstrated in intestinal-muscular cells from *C. elegans*, where extracellular acidification occurs due to activation of an Na^+^/H^+^ exchanger and H^+^ activates a Cys-loop ionic channel (a specific proton receptor) that ultimately produces a muscle contraction [7].

In higher organisms, proton-mediated signaling has been found to mediate fear-conditioned learning, retinal cell activation, cochlear and vestibular afferent neuron synaptic activation, and synaptic transmission in the calyx of Held in the auditory system [8–12].

ASICs form part of the degenerin/epithelial sodium channel (DEG/ENaC) super family [13]. They are voltage-insensitive, cation-selective channels that are mostly permeable to Na^+^ and nonspecifically blocked by amiloride. Seven different subunits, 1a, 1b, 2a, 2b, 3, 4 and 5 (a and b refer to splice variants), derived from 5 genes (*ACCN1–5*) have been identified in mammals. ASICs typically generate transient inward currents in response to increments in the H^+^ concentration of the extracellular media and constitute the sensing element in proton-mediated signaling systems. It is worth to mention that desensitization of the current must probably constitute a protective mechanism in order to avoid sustained activation of the ASIC current in case of persistent acid pH shifts.

**Figure 1:**
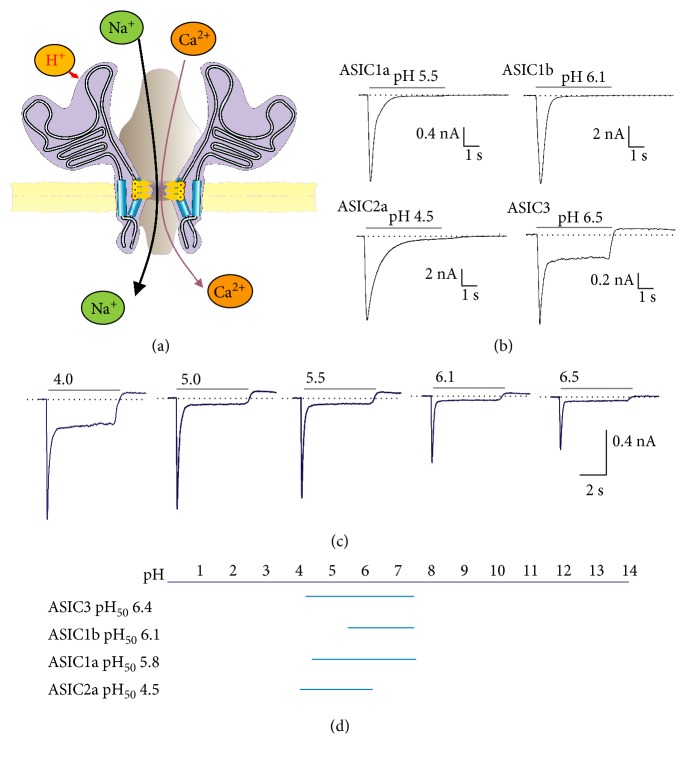
ASIC structure and properties. In (a) scheme of the ASIC trimer. Current is activated by H^+^ and carried by Na^+^ and in lower proportion by Ca^2+^. The increase in intracellular Na^+^ and Ca^2+^ concentrations caused by ASIC current may activate various intracellular messenger systems. In (b), typical ASIC currents obtained from homomers of ASIC subunits transfected in Chinese hamster ovary (CHO) cells [229]. It is worth to note the sustained component of ASIC3 current. In (c), ASIC currents elicited by different pHs in isolated neurons from the rat spiral ganglion [11]. In (d), every subunit has a typical half-activation pH. The various isoforms of ASIC confer the heteromeric channels a larger span of pH responsiveness. Calibration of currents is in nanoampere (nA) versus time in seconds (s).

Each ASIC subunit consists of two transmembrane domains (TM1 and TM2), a large cysteine-rich extracellular loop and short intracellular N- and C-termini (Figure 1(a)) [14, 15]. ASICs are widely expressed by many tissues, either in the nervous system or outside the nervous system (see Table 1 for a summary of the distribution and functions of ASICs).

Most ASIC subunits aggregate and form heteromultimers, generating diverse proton-gated channels that act as acid sensors spanning a large pH range [16, 17]. ASIC4 and ASIC5 are the least studied of these proton-gated channels and are considered to be orphan subunits [18, 19]. Both of them, however, are expressed in the central nervous system.

The crystal structure of the chicken ASIC1a protein demonstrated that functional ASICs are trimeric assemblies [14, 15]. Recent evidence has put this into question; however, data from biochemical studies suggest that a tetrameric conformation of ASICs is also possible [20].

ASIC1a homomers and ASIC1a-ASIC2b heteromers have higher Ca^2+^ permeability; thus, sustained activation of these channels may raise intracellular Ca^2+^ concentrations in neurons [21–23], although recent evidence indicates that the Ca^2+^ permeability of ASICs is of marginal relevance [24].

The function of ASICs depends on their heteromeric composition. For example, the ASIC3 subunit is not expressed by neurons in the cortex or any brain nuclei but is expressed in a transgenic mouse model in which ASIC3 expression is induced in the whole brain, leading to impairment of ASIC-related behaviors, such as fear conditioning. This action may be due to changes that are produced in the biophysical properties of brain ASICs, indicating that the subunit composition and current kinetics are critical for the integration and processing of ASIC-related behaviors [25].

ASICs typically generate transient inward currents that mostly desensitize, although specific channels show some level of sustained acid-dependent current (Figures 1(b) and 1(c)) [17, 26]. Mild or slow acidification may result in steady-state desensitization, even at pH 7.4, and a significant channel population becomes desensitized [27].

The precise concentration of protons required to induce channel activation varies between subunits. Typically, in homomeric channels, the most sensitive subunit is ASIC3, which has a half-activation pH (pH_50_) of 6.4, and ASIC1b, which has a pH_50_ of 6.1. ASIC2a is less sensitive and has a pH_50_ of 4.5, although its pH sensitivity could change because it is subject to endogenous neuromodulators [17, 28]. The set of different ASIC subunit sensitivities allows to discriminate extracellular pH changes from a very acidic pH (approximately 4.0) to a pH of up to 7.8 [17, 29], thus covering a significant range of pH that are relevant for biological processes (Figure 1(d)).

Amiloride, di- and trivalent cations (Pb^2+^ and Gd^3+^) and toxins from anemones, tarantulas, and snakes are examples of molecules that act on ASICs that have become the focus of intensive pharmacological research (see Tables 2 and 3 for a summary of ASIC modulator molecules).

## 2. ASICs in Neurodegenerative Disease

### 2.1. Multiple Sclerosis

Multiple sclerosis (MS) is a chronic autoimmune inflammatory disease of the CNS, whose pathophysiological process involves demyelination and axonal degeneration [30].

In experimental autoimmune encephalomyelitis (EAE), a mouse model of MS, it was found that the *ASIC1* knockout mice showed reduced clinical deficits and axonal degeneration compared with wild-type mice. Furthermore, amiloride produced significant protection in animals with EAE [31].

ASIC1a activation triggers the intracellular accumulation of Na^+^ and Ca^2+^, and previous studies have shown that excessive accumulation of these ions is involved in neuronal degeneration and inflammatory processes in MS [31]. ASIC1 was found to be upregulated in axons and oligodendrocytes in EAE animals, and coincidently, in patients with active MS, a correlation was demonstrated between increased ASIC1 expression and axon injury markers [32]. Moreover, amiloride administration (ASIC-unspecific blocker) attenuated myelin and neuronal damage in animal models as well as in a cohort of MS patients, indicating that amiloride is neuroprotective and could be added to the pharmacological scheme in patients with MS [33].

### 2.2. Parkinson's Disease

Parkinson's disease (PD) is a disabling disease that is characterized by motor impairment, development of Lewy bodies (a pathological hallmark), and progressive loss of dopaminergic neurons in the *substantia nigra* [34].

Neurons in the *substantia nigra* express at least ASIC1a [35, 36]. In a mouse model of PD induced by 1-methyl-4-phenyl-1,2,3,6-tetrahydropyridine (MPTP), amiloride and psalmotoxin-1 (PcTx1; see Table 2) protect neurons from degeneration [36].

Furthermore, mutations of the *Parkin* gene or a lack of endogenous *Parkin* protein produces abnormal ASIC currents and dopaminergic neuronal injury, suggesting that ASIC activity may play a significant role in PD physiopathology [37].

Additionally, paeoniflorin, the principal active ingredient extracted from the root of *Paeoniae alba*, a natural product used in traditional medicine for the treatment of neurodegenerative disorders, blocks ASIC current and also has a neuroprotective effect in PD patients. Paeoniflorin reduces acidosis-induced accumulation of *α*-synuclein (the major component of Lewy bodies); this latter effect could be linked to the inhibition of ASICs, most likely ASIC1a [38].

### 2.3. Epileptic Seizure Activity

During and following seizures, great quantities of lactic acid and glutamic acid are released into the extracellular space, causing a significant fall in pH that activates ASICs [39–41]. In animals treated with pilocarpine to induce a *status epilepticus*, a decrease of ASIC2b mRNA in all hippocampal areas and of ASIC1a mRNA in the CA1-2 was found [42].

Other studies showed that amiloride has anticonvulsant effects *in vivo*, suggesting that ASICs activation might have a proconvulsant potential [43–45]. Amiloride also blocks other ion channels (such as ENaC and T-type Ca^2+^ channels) and membrane exchangers; thus, the action of amiloride is not directly attributable to ASICs blockade.

By contrast, Ziemann and colleagues found that ASIC1a expression is higher in GABAergic interneurons than in excitatory neurons and showed that kainate-induced seizures were longer and more severe in *ASIC1* knockout mice, although it did not affect the seizure threshold [46]. Consistent with the proposal that ASICs participate in ending seizures, the loss of ASIC1a reduced postictal depression [46].

Although ASIC3 expression in the brain is considered to be low or null, some reports described its expression in inhibitory GABAergic interneurons and glial cells [47]. Blocking ASIC3 with its specific antagonist APETx2 in pilocarpine-induced or pentylenetetrazole- (PTZ-) induced seizures shortened the latency to seizure and increased the incidence of generalized tonic-clonic seizures compared to the control group, indicating, as suggested for ASIC1a, that ASIC3 could participate in seizure ending [47].

Evidence also indicates that seizure-induced oxidative stress enhanced expression of the ASIC2a-containing channels that contribute to hyperexcitability, excitotoxicity, and eventually spontaneous seizures. Inhibition of ASICs was neuroprotective in the acute phase after seizure activity [48].

### 2.4. Ischemic Neuronal Injury

During a stroke, the disruption of blood flow to the brain deprives cells of energy and disturbs the cell's ionic homeostasis. Under these conditions, hypoxia enhances anaerobic glycolysis, resulting in the buildup of lactic acid and subsequent tissue acidosis [49, 50]. The extracellular pH in the brain typically drops to values below 6.5 or less during ischemia under normoglycemic conditions, activating ASIC currents [51, 52].

ASICs activation seems to play a fundamental role in acidosis-mediated neuronal injury. ASIC1a activation may trigger membrane depolarization, driving Ca^2+^ influx via ASIC1a homomers or ASIC1a-ASIC2b heteromers, voltage-gated Ca^2+^ channels, and NMDA receptors [21, 22, 53–57].

In cultured mouse and human cortical neurons, activation of ASICs induces glutamate receptor-independent neuronal injury that is inhibited by specific ASIC1a blockade and by *ASIC1* gene knockout [21, 57]. Intracerebroventricular injection of PcTx1 (ASIC1a blocker/inhibitor) in animal models of brain ischemia reduced the infarct volume by up to 60%. Protection by an ASIC1a blockade has an efficacy time window of approximately 5 hours, and the protection persists for at least 7 days [21, 54].

Furthermore, an *ASIC1* gene knockout leads to significant neuroprotection in mice, and the reduction of ASIC1a expression contributes to neuroprotection as elicited by ischemic preconditioning and postconditioning in rats [21, 58]. Increasing ASIC1a surface expression, for example, through inhibition of ASIC1a internalization, exacerbates acidosis-induced neuronal injury [59]. In a model of global ischemia, the ASICs inhibitor amiloride, but not the NMDA receptor blocker memantine, reduced brain damage indicating that in some models of brain ischemia, ASICs may play a larger role than glutamatergic NMDA receptors in the mediation of neuronal injury [60]. The protective effect of ASICs inhibition is additive to that of NMDA receptor inhibition, thus indicating that they take place by different mechanisms [61].

After an ischemic insult, AMPA receptor plasticity exacerbates excitotoxic damage in the hippocampal region, particularly the increased expression of Ca^2+^ permeable GluA2-lacking AMPA receptors (CP-AMPAR), which may play a significant role in postischemic neuronal cell death. In hippocampal slice cultures exposed to oxygen-glucose deprivation and in hippocampal pyramidal neuron cultures exposed to acidosis, it was found that ASIC1a activity promotes the expression of CP-AMPAR and of anoxic long-term potentiation, but ASIC1a inhibition confers neuroprotection [62]. In contrast, an opposite effect indicating that the ASIC1a deletion increases CP-AMPAR expression in the nucleus accumbens of the ventral striatum was also reported [5]. This discrepancy may be because ASIC1a could differentially regulate the expression of CP-AMPAR in a specific tissue manner.

Other ischemia-associated factors, such as arachidonic acid and lactate, endogenous polyamines, large dynorphin, and nitric oxide, also exacerbate acidosis-mediated neuronal injury and ischemic damage [56, 63–65]. These factors may act by enhancing the ASIC current or slowing its desensitization. For example, spermine, which is an endogenous polyamine found at high concentrations in the brain, shifts the steady-state inactivation of ASIC1a and potentiates ischemia-induced injury in the brain during stroke [66], showing that steady-state desensitization is a critical factor that can affect the degree of acid-evoked neuronal damage through ASIC1.

Interestingly, three compounds, puerarin, sophocarpine, and ginsenoside-Rd (found in several traditional Chinese preparations), as well as the flavonoid quercetin, protect against damage caused by middle cerebral artery occlusion. These compounds were found to reduce the current amplitude of ASIC1a, increase channel desensitization, or decrease ASIC1a expression [67–69].

Altogether, these findings support the participation of ASICs in excitotoxic neuronal injury and suggest a new pathophysiological model for ischemic brain injury in which extracellular acidification produces an over activation of the ASIC current. Thus, ASICs constitute a new therapeutic target for the treatment of ischemia-induced neuronal damage.

### 2.5. Spinocerebellar Ataxia

Spinocerebellar ataxias (SCAs) are a group of autosomal dominant progressive neurodegenerative disorders that display complex clinical and genetic heterogeneity. Spinocerebellar type 1 ataxia (SCA1) primarily affects the brainstem, spinocerebellar tracts, and cerebellar Purkinje cells (PC). Patients with SCA1 develop progressive ataxia accompanied by bulbar and pyramidal symptoms [70]. Mutations of the ataxin-1 gene are responsible for the disease, and induction of the mutation in mice produces many of the clinical features observed in SCA1 patients. The exact mechanism of PC and of spinocerebellar tract cell loss remains unclear. Currently, there are no specific treatments for SCA1.

Excitingly, ataxin-1 transgenic mice induced in an *ASIC1a* knockout mouse background demonstrated that deletion of the *ASIC1a* gene suppresses the SCA1 disease phenotype, improving the motor deficit and decreasing PC degeneration. This shows that ASIC1a may be a mediator of SCA1 pathogenesis and that targeting ASIC1a could be a novel approach to treat SCA1 [71].

SCA3 ataxia (the most common one) results from a CAG-trinucleotide expansion in the coding region of the *ATXN3* gene, leading to an expanded polyglutamine (polyQ) sequence within the Ataxin-3 protein. Using the fruit fly *D. melanogaster* as a model, it was demonstrated that downregulating the *Nach* gene (an ortholog of ASICs in the fly) mitigates SCA3 pathogenesis, indicating that ASICs may be involved in the pathophysiology of SCA3 [72].

### 2.6. Malignant Glioma

Malignant gliomas, the most common subtype of primary brain tumors, are aggressive, highly invasive, and neurologically destructive tumors that are considered to be among the deadliest of human cancers [73]. ASIC1a is extensively expressed in malignant glial cells. Amiloride- and PcTx1-sensitive cation currents in human glioblastoma are produced by mixed ASIC and ENaC components, including ASIC1 and ASIC2. Inhibition of ASIC1 conductance by PcTx1 and by the amiloride analog, benzamil, decreases the glioma growth rate and cell migration as well as arrests the cell cycle [74–78]. *ASIC1a* knockdown models show a significant inhibition of glioblastoma cell migration [77].

By contrast, increasing surface expression of the ASIC2 subunit suppressed the proliferation and migration of glioblastoma cells [75]. This last result suggests that the role of ASIC subunits in the pathophysiology of glial cancer is complex and that no straightforward intervention seems to be feasible; thus, further knowledge of the role of ASICs in neoplastic development is required to develop a translational use of ASIC blockers or enhancers in this pathology.

## 3. ASICs in Inflammatory Processes and Pain

The inflammatory process implies activation of immune cells and release of a cocktail of chemical mediator known as “inflammatory soup.” The inflammatory reactions are self-limited by the elimination of the cause [79, 80]. ASICs may be modulated by various components of the inflammatory soup, including NGF, 5-HT, and bradykinin, among others [81, 82]. In addition, the natural polyamines agmatine and arcaine may activate the ASIC3 subunit (the concentration of polyamines can increase to up to 1 mM in inflamed tissues) [83], while spermine enhances ASIC1a activity by slowing its inactivation and accelerating its recovery from desensitization [66]. Indeed, intraplantar injections of 2-guanidine-4-methylquinazoline (GMQ) in wild-type mice cause marked pain-related behaviors that are abolished in *ASIC3* knockout mice [82].

It has been proposed that during inflammation, activation of ASICs is essential in nociception transduction and production of painful sensations. In sensory neurons, inflammation induces an increase in the ASIC current and its expression in the cell membrane, leading to an increase in neuronal excitability [82].

Microglia have been found to express ASICs, and stimulation of microglia with lipopolysaccharides leads to an increase in ASIC1 and ASIC2a expression as well as the release of inflammatory cytokines [84], demonstrating the role that ASICs play in neural sensitization during inflammatory processes.

### 3.1. Primary Inflammatory Pain

The role of ASICs in primary inflammatory pain has been investigated using various *in vitro* and *in vivo* experimental models [66, 85–88]. Most of the ASIC subtypes are expressed on nociceptive primary sensory neurons, where they seem to play a significant role in pain transduction [3, 89]. Cutaneous pain produced by low pH solutions (at least over pH 6.0) is likely due to ASIC activation [90, 91].

Compounds inhibiting ASICs display an analgesic effect in animal models of pain [92], whereas those activating ASICs elicit pain behavior [85, 86, 88, 93], supporting a role for ASICs in the transduction of cutaneous pain.

During inflammation, the pH value of the local area is always lower than the physiological pH, ranging from pH 5.5 to 7 [94]; this pH drop is sufficient to activate ASICs. Indeed, inflammation induces a marked increase of ASICs expression in primary sensory neurons, and nonsteroidal anti-inflammatory drugs (NSAIDs) attenuate the ASIC current [95]. Furthermore, in isolated dorsal root ganglion (DRG) neurons, a mixture of proinflammatory mediators, such as nerve growth factor, serotonin, interleukin-1, and bradykinin, increases the number of neurons expressing ASIC as well as their current density [82]. Bradykinin and serotonin act on ASICs through an indirect intracellular signaling pathway involving protein kinase C. Bradykinin activates bradykinin B1 and B2 G protein-coupled receptors (GPCR) [96], while serotonin acts on the GPCR 5-HT_2_ [97]. B1, B2, and 5-HT_2_ receptors are constitutively expressed in sensory neurons, and their activation has been associated with inflammatory hyperalgesia. The binding of bradykinin and serotonin to their receptors induces phospholipase C (PLC) stimulation through heterotrimeric G proteins (G_q/11_) [96, 98, 99]. PLC cleaves phospholipid phosphatidylinositol 4,5-bisphosphate (PIP2) into diacyl glycerol (DAG) and inositol 1,4,5-trisphosphate (IP3). IP3 releases Ca^2+^ from the internal Ca^2+^ stores in the endoplasmic reticulum, and DAG activates PKC. ASICs have a PDZ-binding domain at their C-termini; its interaction with PDZ-containing proteins regulates surface expression and activity of ASICs [37, 99]. The protein C-kinase (PICK1) colocalizes with ASICs, interacting directly through the PDZ-binding domain [100]. Therefore, the PKC signaling pathway may be involved in the enhancement of the ASICs mediated by inflammatory mediators.

Serotonin also directly binds to the extracellular domain of ASIC3 to increase its current [101]. The enhancing action of serotonin occurs in the sustained component of the ASIC3 current, which is particularly important for its pain-mediating effect [102–104]. Interestingly, serotonin acts not only on ASIC3 homomeric channels but also on heteromeric channels composed of ASIC3-ASIC1a or ASIC3-ASIC1b subunits [101].

Other inflammatory stimuli, such as ATP, lactic acid, arachidonic acid, nitric oxide (NO), agmatine, and hypertonicity, are able to enhance the proton-induced ASIC current. ATP can increase the pH sensitivity of ASIC3, and it has been proposed that this phenomenon of “sensitization” involves a protein assembly of P2X purinergic receptor and ASICs [57, 63, 85, 105, 106].

NO can potentiate the activity of ASICs in DRG neurons and Chinese hamster ovary (CHO) cells expressing ASIC subunits; this potentiation is probably due to the oxidation of cysteine residues of the channels. Additionally, topical application of the NO donor glyceryl trinitrate significantly increased acid-evoked pain in human volunteers without affecting their heat or mechanical pain threshold [107]. In the central nervous system, ASIC potentiation by NO aggravates acid-induced cell death during mild or moderate acidosis [55].

Histamine and histamine agonists were shown to potentiate ASIC currents in transfected CHO cells, apparently by specifically binding to the acid pocket of ASIC1a subunits [108]. Our research group found that histamine also potentiates ASIC currents in DRG neurons isolated from the rat, thus contributing to hypersensitivity in inflammatory conditions.

The ASIC currents in rat DRG neurons are produced by ASIC1 and ASIC3 [85, 109, 110]. The anemone toxin APETx2, which blocks ASIC3-containing channels, has potent analgesic effects after local application in rodent cutaneous acidic and inflammatory pain [111, 112].

ASIC3 also participates in the maintenance of subacute primary hyperalgesia (an increased response to noxious stimuli at the site of injury) in the case of cutaneous inflammation [113]. *ASIC3* knockout mice fail to develop secondary hyperalgesia (an increased response to noxious stimuli outside the site of injury) induced by either repetitive acid injections into muscle [114], muscle inflammation [114], or knee inflammation [115].

Intrathecal injection of the ASIC1a blocker PcTx1 attenuates acute pain responses as well as pain behaviors in chronic inflammatory and neuropathic models. This is probably due to activation of the encephalinergic system secondary to ASIC1a blockade, but the precise mechanism has not yet been defined [88]. Additionally, a recently identified blocker of ASIC1a from the venom of the black mamba (*Dendroapsis polylepis*) named mambalgin-1 attenuated a variety of pain behaviors when administered centrally [86].

Injection of coral snake toxin MitTx, a potent activator of ASIC1 into the skin of the hind paw of a mouse produces a nociceptive behavior that is missing in *ASIC1a* knockout mice [92]. Similarly, mambalgin-1 (the ASIC1 and ASIC2 blockers) administered via intraplantar injection in mice attenuates both acute thermal nociception and inflammatory hyperalgesia, an effect that is lost in *ASIC1b* knockdown [86]. Finally, two clinical studies successfully used amiloride or a nonsteroidal anti-inflammatory drug to inhibit acid-evoked pain in human skin [91, 92].

Additionally, the aminoglycosides streptomycin and neomycin, which were shown to inhibit proton-gated currents in rat DRG neurons and reduce their action potential response to an acidic stimulus, have a significant analgesic action when administered locally in a model of inflammatory pain [87, 116]. Streptomycin also reduces the GMQ-induced nociceptive behavior, indicating that aminoglycoside antibiotics produce analgesia due in part to the inhibition of ASICs activation in sensory neurons [116].

Although diverse evidence attests to the role of ASICs in nociception, conflicting results from studies of knockout and transgenic mice exist. In acute pain paradigms, *ASIC3−/−* mice were hyperalgesic to high-intensity thermal, mechanical, and acid stimuli [117, 118]. Similar results were found in transgenic mice overexpressing dominant-negative *ASIC3* [119].

### 3.2. Migraine

Migraine is one of the most common neurological disorders and a chronic pain condition that is usually accompanied by a variety of symptoms, including aura, nausea, vomiting, photophobia, and phonophobia. Although the exact pathophysiology of migraine headaches is still partially unknown, activation of the meningeal sensory neurons is likely required [120, 121].

In 80% of trigeminal afferent neurons originating from meninges, ASIC-like currents can be evoked at pH 6.0, and over 50% are also responsive to pH 7.0 [121]. In animal models of migraine where low pH stimuli (from 5 to 6.4) were applied directly to the dura mater of awake animals, the acidic pH produced headache-like behavior that was blocked by amiloride or APETx2 (pH 6.0) [122].

Cortical spreading depression (CSD) is a neuronal process that linked to migraine and consists of brief neuronal excitation followed by a longer-lasting depression of activity. A CSD event propagates across the cortex in a wave-like mode. CDS is thought to be linked to migraine with aura because during this phase, changes in vision, particularly the movement/expansion of geometric shapes or scintillating scotomas, occur and can be mapped as electrical changes in the visual cortex consistent with CSD propagation. Whether CSD participates in other phases of migraine is unclear [40, 123]. Amiloride and PcTx1 block CSD and inhibit trigeminal activation in migraine models *in vivo* [124]. Intriguingly, there was no effect of ASIC blockers against CSD evoked by high K^+^, suggesting that ASICs may not contribute to all types of CSD. In a small clinical study, amiloride reduced both the aura frequency and headache severity [124].

### 3.3. Gastrointestinal Pain

ASICs (ASIC1, ASIC2, and ASIC3) are expressed by the peripheral axons of vagal and spinal afferent neurons. Retrograde tracing studies indicate that 75% of the nodose ganglion neurons and 82% of the DRG neurons projecting to the rat stomach express ASIC3-like immunoreactivity [125]. In mouse thoracolumbar DRGs, ASIC3 is expressed in 73%, ASIC2 in 47%, and ASIC1 in 30% of the neurons projecting to the mouse colon [126].

ASIC3 plays a major role in inflammatory hyperresponsiveness to gastric acid as it may occur in gastritis and peptic ulcer disease. Disruption of the *ASIC3* gene abolished the effect of gastritis and enhanced gastric acid-evoked expression of c-Fos in the brainstem. Conversely, *ASIC2* gene knockout does not alter inflammatory hyperresponsiveness but enhances the medullary c-Fos response to the gastric acid challenge of the stomach [127]. *ASIC3−/−* mice have markedly reduced visceral mechanosensitivity compared to control animals and *ASIC1−/−* or *ASIC2−/−* mice [128], thus indicating the significant role of ASIC3 in gastrointestinal nociception.

The nonselective ASICs blocker benzamil produces a partial attenuation of the mechanosensitivity of gastroesophageal afferents, but its effect is more significant in colonic afferents [128]. The differential role of ASIC3 in the upper and lower GI tract indicates that this channel may serve as a key target for modulating GI nociception.

It is worth noting that dietary intake and bacterial metabolism may generate high concentrations of polyamines in the gut that may potentiate at low concentrations or directly activate ASIC currents [129]. Noteworthy also, some intestinal parasites, such as *Echinococcus granulosus*, produce and release peptides that may inhibit ASIC currents [130], thus producing an antinociceptive action and also probably an action modulating the dendritic cell response activated by acid which requires ASICs activation [131] Thence, the ASIC inhibition may constitute a mechanism by which parasites mitigate the nociception and immune response from the host, facilitating parasite infestation.

### 3.4. Cardiac Pain

Cardiac afferent neurons express ASIC3 that are activated by mild acidification during ischemia [132, 133]. The acid-gated currents from *ASIC3−/−* cardiac afferents match the properties of ASIC2a, and currents from *ASIC2−/−* cardiac afferents match the properties of ASIC3 [133], thus demonstrating that ASIC currents in myocardial afferents are due to ASIC3-ASIC2 heteromers. ASIC3 seems to be the sensor of myocardial acidity that triggers cardiac pain, thus constituting a potential pharmaceutical target for treating angina pectoris [133].

Regrettably, not all ASIC-blocking drugs are useful for treating cardiac pain during ischemic attacks; it has been shown that amiloride, although, reducing the peak ASIC current in cardiac sensory neurons, is also able to increase the sustained component [104], which is predominantly expressed in ASIC3 and important for the transduction of chemical stimuli and nociception (Figure 1(b)). In contrast, ligustrazine, a compound extracted from the roots of *Ligusticum chuanxiong*, attenuated ASIC currents in DRG neurons and in CHO cells transfected with ASIC cDNAs [134]. In a rat model of angina, ligustrazine and ASICs inhibitor A-317567 also reduced the cardiac ischemia-induced electrical dysfunction and infarct size. Thus, inhibition of ASICs by ligustrazine may explain the beneficial effects of the drug in patients with ischemic heart disease and angina [134].

### 3.5. Postoperatory Pain

Postoperative pain is a common clinical condition produced by multiple processes, including tissue damage, secondary inflammation, and nerve damage caused by tissue manipulation during surgery [135]. Pain can be acute or chronic and produces a series of physiological consequences, including activation of the “stress response,” which broadly affects various tissues in patients [136].

In a rat model of postoperative pain, high levels of ASIC-type currents (~77%) were found in sensory neurons innervating the hind paw muscles, with a high prevalence of ASIC3-like currents. Pharmacological inhibition of ASIC3 with APETx2, or *in vivo* knockdown of *ASIC3* by interfering RNA, led to a significant reduction of postoperative spontaneous, thermal, and postural pain behavior. A single intra-operative application of APETx2 was an effective analgesic for 24 hours after surgery [111].

### 3.6. Muscular Pain

The role of ASICs in muscle pain has been studied in inflammatory and noninflammatory models [137, 138]. In noninflammatory pain, an intramuscular acidic saline injection produces nociceptive behavior [139, 140]. Intramuscular acid-induced pain was seen in *ASIC1−/−*, but not in *ASIC3−/−* mice, although *ASIC1−/−* mice developed secondary mechanical hyperalgesia of the paw; however, this response was completely abolished in *ASIC3−/−* mice [114]. Similarly, blockade of ASICs with amiloride or with the ASIC3 antagonist APETx2 prevents the development of hyperalgesia [112, 140]. ASICs inhibition 24 hours after a second intramuscular acid injection, at a time when hyperalgesia is well established, had no effect on muscle or cutaneous hyperalgesia. In patch clamp recordings from DRG neurons 24 h after the second acid injection, neurons show no changes in responsiveness to acidic pH stimuli, suggesting that long-lasting hyperalgesia is independent of ASICs activation [137].

In human subjects, infusion of an acidic buffer into the anterior tibialis muscle results in local pain at the injection zone and also produces a referred pain at the ankle; additionally, subjects report hyperalgesia to pressure pain at the site of infusion and at the ankle (secondary hyperalgesia) [141].

In adult mice, knockdown of *ASIC3* in DRG innervating muscle with microRNA (miRNA) prevents the development of both paw and muscle hyperalgesia in mice with muscle inflammation [142]. Twenty-four hours after carrageenan-induced muscle inflammation in mice, the mRNA of ASIC2 and ASIC3 (but not ASIC1) in lumbar DRG neurons increases bilaterally [143]. Additionally, in DRG neurons that innervate muscle, there are enhanced ASIC-like currents under an acidic pH [139]. The study also shows that the nonselective ASIC inhibitor A-317567 can reverse both primary and secondary hyperalgesia.

Rescuing ASIC3 expression in primary afferent fibers that innervate muscle in *ASIC3* knockout mice restores the mechanical hyperalgesia of the paw [115], whereas downregulation of ASIC3 in muscular sensory fibers in wild-type animals prevents the development of inflammatory hyperalgesia [142].

ASIC3 was involved in the transition from acute to chronic pain in a mouse model of fibromyalgia induced by intramuscular acid injections. Inhibition of ASIC3 with APETx2 abolished hyperalgesia at the time of the first acid injection and prevented the induction of chronic hyperalgesia by a subsequent acid injection (five days later). It was also shown that *ASIC3−/−* mice did not develop mechanical hyperalgesia after repeated acid injections [144, 145].

## 4. Conclusion

The etiology of neurodegenerative diseases is varied and probably multifactorial, but these diseases share common processes, such as accumulation of misfolded proteins or metabolic alterations leading to damage to specific neuronal populations as well as chronic inflammation; therefore, two seemingly distant processes, such as neurodegeneration and neuroinflammation, can be causally related and share one or more similar pharmacological targets [146].

Neurodegenerative disorders are associated with different processes such as neurovascular disintegration, defective blood-brain barrier function, and microvascular dysfunction [147]; these processes cause a decrease in brain flow that leads to a reduction in the oxygen and nutrient supply to the brain, in addition to causing a decrease in the extracellular pH that may lead to apoptosis [148], protein misfolding [149, 150], excitotoxicity [151, 152], and neurodegeneration [153, 154]. In this context, several noninvasive methods have been developed to measure pH with high spatial and temporal resolution in both clinical and preclinical studies of neurodegenerative disorders. In fact, it has been suggested to use pH measurements by means of magnetic resonance spectroscopy as a potential biomarker of neurodegeneration [155–157].

Several studies have shown that ASICs play a significant role in inflammatory processes, as well as in neurodegenerative diseases, such as Parkinson's disease, multiple sclerosis, and cerebrospinal ataxia, among others. The use of several techniques, including electrophysiology, molecular biology, genetics, biochemistry, and in silico analysis, has produced a large amount of knowledge indicating the salient role of ASICs in the physiology and pathophysiology of inflammatory and degenerative diseases.

Because the ASICs show a significant desensitization to pH stimuli, they cannot remain activated during long-term pH changes, or in any case, it remains a sustained component of the current, but its magnitude is low and in the long term must probably completely cancelled; thus explaining why sustained acidosis is necessary but not sufficient to damage the SNC. So, as in many other physiological and pathological processes, a combination of factors is essential for its development. Thence, ASICs activation seems necessary but not a sufficient cause to produce neurodegeneration. The production of ASICs modulator agents such as sulfhydryl-containing molecules dithiothreitol (DTT) and glutathione [158], among others, may shift the pH of ASICs toward more neutral pH and slow channel desensitization, thus significantly increasing the inward sodium current passing through the ASICs and contributing to enhance the acid-induced tissue damage.

Inflammation is a necessary and evolutionarily conserved response to harmful stimuli that produce tissue damage or degeneration as well as to various pathogens that invade the host. The inflammatory process leads to the release of numerous mediators, including purines, prostaglandins, bradykinins, histamine, serotonin, nerve growth factor (NGF), cytokines, and protons (among others) [159]. The low pH levels found in inflamed tissue led to the hypothesis that local acidosis may contribute to pain and hyperalgesia. ASICs are involved in nociceptive transduction and DRG neuronal sensitization, thus constituting a new, potentially significant target for the treatment of pain and hyperalgesia in diseases associated with inflammation.

Pharmacologically, the substances that modulate ASICs have grown significantly and now include many synthetic inhibitors as well as various organic molecules obtained from animals, vegetables, and even endogenous ones [160]. The increase in pharmacological, physiological, and pathophysiological processes that are mediated by ASICs opens new perspectives for the synthesis of pharmacological tools that may contribute to the armamentarium against neuroinflammatory diseases, some of the most challenging health problems faced in modern times. Furthermore, there is a trend to search for multitarget molecules that may reach an efficient neuroprotective effect [161].

## Figures and Tables

**Table 1:** Distribution and functions of ASICs.

Subunit	Distribution	Physiology	Pathophysiology
1a	Brain [29], spinal cord [117], DRG [3], TG [128], NG [132], cochlear and vestibular neurons [9, 11, 162], retina [8], astrocytes [163], lung epithelial cells [164], vascular smooth muscle cells [165], microglia [84], bone [166], taste receptor cells [167]	Synaptic plasticity [168], learning and memory [168], fear conditioning [169], visual transduction [8], visceral mechanoperception, primary muscle hyperalgesia [143], apoptosis [170], chondroprotection and bone resorption [171]	Pain [88], inflammation [172], migraine [124], epilepsy [46], hepatic fibrosis [173], multiple sclerosis [32], Parkinson [174], Huntington [175], anxiety [176], depression [177], growth and migration of gliomas [178], excitotoxicity [179]

1b	DRG [3], immune cells [31], taste receptor cells [180], carotid body [181], and cochlear hair cells (stereocilia) [182]		Pain [118], inflammation [31, 143], cancer [78]

2a	Brain [117], DRG [3], NG [183], spinal cord [117], retina [8], cochlear and vestibular neurons [9, 11], astrocytes [163], microglia [84], bone [166], lung epithelial cells [164], vascular smooth cells [165], taste receptor cells [167], carotid body [181]	Visual transduction [8], detection of sour taste [184], mechanosensation [185], arterial baroreceptor reflex [186]	Inflammation [84], ischemia [187], migration of gliomas [75]

2b	Brain [29], spinal cord [188], DRG [3], NG [183], JG [183], cochlear neurons [11], retina [189], and taste receptors [190]	Integrity of retina [8], modulator of ASIC1a, ASIC1b, ASIC2a, and ASIC3 [23]	Inflammation [84], gastrointestinal pain [128]

3	DRG [3], TG [128], cochlear and vestibular neurons [9, 11], vagal and glossopharyngeal ganglia [183], brain [29], spinal cord [188], retina [191], taste receptors [167], astrocytes, microglia [84], testis [192], chondrocytes and synoviocytes [115], adipocytes [163], immune cells [31], lung epithelial cells [164], bone [166], cartilage [166], teeth [193], vascular smooth muscle [165], and carotid body [181]	Chemoreception [181], skin mechanosensory [139], auditory and visual processing [191], mechanosensory of the intestinal tract [128]	Pain [85], inflammation [85], epilepsy [47], migraine [122], gastrointestinal pain [128], cardiac pain [63], postoperatory pain [109], secondary mechanical hyperalgesia [142]

4	Brain [194], spinal cord [117], pituitary gland [195], immune cells [31], and retina [196]	A possible function is to decrease the amount of functional ASICs in the plasma membrane and as a regulator of pain [197]	Pain [197]

DRG: dorsal root ganglia; TG: trigeminal ganglia; NG: nodal ganglia; SG: spiral ganglia; JG: jugular ganglia.

**Table 2:** Exogenous modulators.

	Compound	Effect	Subunit
Toxins from venoms	PcTx1	↑ the affinity of the proton, desensitizes the channel [198]	ASIC1a, 1a-2b
	Hi1a	Stabilizes the close state of the channel, impeding the transition into a conducting state [199]	ASIC1a
	APTx2	↓ ASIC3 and ASIC3 heteromers [200]	ASIC3
	MitTx	↑ increase the sensitivity of ASIC2a to protons and activate ASIC1a, 1b, and 3 [92]	ASIC1a, 1b, 2a, and 3
	Mambalgins	↓ potent, rapid, and reversible inhibitor of ASICs [86].	ASIC1a, 1b, 1a-2a, 1a-2b, and 1a-1b
	Ugr 9-1	↓ ASIC3 current, including sustained component [201]	ASIC3
	PhcrTx1	↓ ASICs in nDRG [202]	All subunits
	*α*-Dendrotoxin	↓ ASICs in nDRG [203]	?

Vegetal compounds	Thalassiolin B	↓ ASICs in nDRG [204]	Currents with desensitization < 400 ms
	Sevanol	↓ ASIC1a and 3 (including sustained component) [205]	ASIC1a and 3
	Gastrodin	↓ ASICs in nDRG [206]	All subunits
	Puerarin	↓ ASICs in rat hippocampal neurons and homomers [67]	ASIC1a
	Chlorogenic acid	↓ ASICs in nDRG [207]	All subunits
	Morphine	↓ ASICs in nDRG [208]	All subunits
	Paeoniflorin	↓ ASICs in PC12 cell line [38]	ASIC1a
	Ligustrazine	↓ ASICs in nDRG and ASIC heteromers [134]	ASIC1a, 1b, 2a, and 3

	Cannabinoids	↓ ASICs in nDRG [209]	All subunits

NSAIDs	Salicylic acid, aspirin, diclofenac, fluribuprofen, ibuprofen, peroxicam	↓ homomers and heteromers of ASIC1a, 2a, and 3 [210]	ASIC1a, 2a, and 3
	CHF5074	↓ ASICs in CA1 pyramidal neurons [211]	ASIC1a

Anesthetics	Tetracaine	↓ homomeric ASICs and ASICs in nDRG [212]	ASIC1a, 1b, and 3
	Lidocaine	↓ ASIC1a and heteromers [213]	ASIC1a and heteromers
	Propofol	↓ ASICs in nDRG [214]	All subunits

Aminoglycosides	Streptomicyn, neomicyn, gentamicyn	↓ ASICs in nDRG [87]	All subunits

Monoamines	9-Aminoacridine (9AA)	↓ ASICs in rat hippocampal neurons and homomers [215]	ASIC1a and ASIC3
	Memantine	↓ ASICs in hippocampal neurons and homomers [215]	ASIC1a, 2a, and 3
	IEM1921	↑ ASICs in rat hippocampal neurons, ↓ ASIC3 current and potentiated the steady state [215]	ASIC1a, ASIC3
	IEM2117	↑ ASICs in hippocampal neurons and homomers [215]	ASIC1a, 2a

Others	Amiloride	↓ unspecific inhibitor of ASIC subunits [13]	All subunits
	4-Chlorophenylguanidine	Activating the channel and increasing proton sensitivity [216]	ASIC3
	GMQ	↑ ASIC3 homomers at neutral pH [82]	ASIC3
	Tetraethylammonium (TEA)	↓ heteromeric ASIC currents [23]	ASIC1a-2b
	4-Aminopyridine (4AP)	↓ heteromeric ASIC currents [217]	ASIC1a-2a and 1a-2b
	A-317567	↓ ASICs in nDRG [93]	All subunits
	Nafamostat mesilate	↓ ASIC currents [218]	ASIC1a, 2a, and 3
	Diarylamidines	↓ ASICs in hippocampal neurons and heteromers [219]	ASIC1a, 1b, 2a, and 3
	Chloroquine	↓ ASICs in retinal ganglion neurons and CHO cells [220]	ASIC1a
	NS383	↓ homomeric and heteromeric channels [221]	ASIC1a, 3, and heteromers
	Omeprazole	↑ the expression of ASIC1a in Caco-2 cells [222]	ASIC1a
	Corticosterone	↑ ASIC1a current in hippocampal neurons [223]	ASIC1a
	Insulin	Regulator of ASIC1a membrane surface expression [224]	ASIC1a, 2a, and 3
	Sulfhydryl compounds	↑ peak current and slow-down channel desensitization [158]	ASIC1a, 1b, 2a, and 3

↓: inhibits; ↑: increases; nDGR: neurons of dorsal root ganglia; NSAIDs: nonsteroidal anti-inflammatory drugs.

**Table 3:** Endogenous modulators.

	Compound	Effect	Subunit
Neuropeptides	Dynorphin A, big dinorphin	↓ decrease proton sensitivity of steady-state inactivation [65]	ASIC1a
	FMRFamide and related mammalian FF amide peptides.	↑ enhance the sustained current and slow down inactivation [225]	ASIC1a, ASIC1b, and ASIC3

Inflamatory mediators	Nerve grow factor (NGF)	↑ ASIC3 expression associated with hyperalgesia [81]	ASIC3
	Bradiquinine	↑ ASIC mRNA levels [81]	ASIC1a, 1b, 2b, and 3
	Serotonin	↑ ASIC3 sustained current [101]	
	Interleucine	↑ ASIC mRNA [81]	ASIC1a, 1b, 2b, and 3
	Arachidonic acid	↑ peak current [106]	
	Nitric oxide (NO donors)	↑ increase ASIC1a, 1b, and 3 current [55, 107]	

Cations	Ca^2+^, Mg^2+^, Cd^2+^, Cu^2+^, Gd^3+^, Ni^2+^, Pb^2+^, Ba^2+^	↓ decrease the ASIC conductance [160]	All subunits
	Zn^2+^	↓↑ dual effect: at low concentration ↓ the current and high concentration ↑ the current [226]	ASIC1a, 1a-2a, and ASIC2a
	NH_4_^+^	↑ activate ASIC current at extracellular pH 7.4 [35]	ASIC1a

Polyamines	Spermine	↑ activity of ASICs by shifting the steady-state inactivation to more acid values [66]	ASIC1a and 1b
	Agmantine and arcaine	↑ activate ASIC3 current [82, 83]	ASIC3

Others	Lactate	↑ ASIC current [63]	ASIC1a and 3
	ATP	↑ pH sensitivity of ASIC3 [105]	ASIC3
	Cl^−^	↑ slow down the rapid desensitization of ASIC1a and maintains tachyphylaxis [227]	ASIC1a
	H_2_O_2_	↓ ASIC1a current [228]	ASIC1a
